# Comparative mathematical modeling of causal association between metal exposure and development of chronic kidney disease

**DOI:** 10.3389/fendo.2024.1362085

**Published:** 2024-05-01

**Authors:** Miaoling Wu, Weiming Hou, Ruonan Qin, Gang Wang, Da Sun, Ye Geng, Yinke Du

**Affiliations:** ^1^ Department of Environmental Health, School of Public Health, China Medical University, Shenyang, China; ^2^ Department of Medical Engineering, Air Force Medical Center, PLA, Beijing, China; ^3^ Experimental and Teaching Center, School of Public Health, China Medical University, Shenyang, China; ^4^ Department of Nephrology, The First Hospital of China Medical University, Shenyang, China; ^5^ Blood Purification Center, The First Hospital of China Medical University, Shenyang, China

**Keywords:** Chronic Kidney Disease, Bayesian kernel machine regression, metal mixtures, machine learning, mediating effect

## Abstract

**Background:**

Previous studies have identified several genetic and environmental risk factors for chronic kidney disease (CKD). However, little is known about the relationship between serum metals and CKD risk.

**Methods:**

We investigated associations between serum metals levels and CKD risk among 100 medical examiners and 443 CKD patients in the medical center of the First Hospital Affiliated to China Medical University. Serum metal concentrations were measured using inductively coupled plasma mass spectrometry (ICP-MS). We analyzed factors influencing CKD, including abnormalities in Creatine and Cystatin C, using univariate and multiple analysis such as Lasso and Logistic regression. Metal levels among CKD patients at different stages were also explored. The study utilized machine learning and Bayesian Kernel Machine Regression (BKMR) to assess associations and predict CKD risk based on serum metals. A chained mediation model was applied to investigate how interventions with different heavy metals influence renal function indicators (creatinine and cystatin C) and their impact on diagnosing and treating renal impairment.

**Results:**

Serum potassium (K), sodium (Na), and calcium (Ca) showed positive trends with CKD, while selenium (Se) and molybdenum (Mo) showed negative trends. Metal mixtures had a significant negative effect on CKD when concentrations were all from 30^th^ to 45^th^ percentiles compared to the median, but the opposite was observed for the 55^th^ to 60^th^ percentiles. For example, a change in serum K concentration from the 25^th^ to the 75^th^ percentile was associated with a significant increase in CKD risk of 5.15(1.77,8.53), 13.62(8.91,18.33) and 31.81(14.03,49.58) when other metals were fixed at the 25^th^, 50^th^ and 75^th^ percentiles, respectively.

**Conclusions:**

Cumulative metal exposures, especially double-exposure to serum K and Se may impact CKD risk. Machine learning methods validated the external relevance of the metal factors. Our study highlights the importance of employing diverse methodologies to evaluate health effects of metal mixtures.

## Introduction

Chronic Kidney Disease (CKD) is a condition that occurs when the kidneys sustain damage and exhibit abnormalities in blood parameters and urine for a duration of more than 3 months. It is also diagnosed when the effective glomerular filtration rate falls below 60% (1). The global prevalence of CKD has been increasing, with a 29.3% rise between 1990 and 2017. This disease carries a substantial global burden, affecting approximately 10% of the adult population and resulting in 1.2 million deaths and 28 million years of life lost annually (2). In China, CKD has emerged as a major health concern, causing significant loss of healthy life expectancy for individuals (3), and imposing a heavy financial and disease burden on the healthcare system.

CKD is a complex condition influenced by both genetic predispositions and environmental factors. Alongside well-known risk factors like aging, diabetes, and hypertension, certain environmental chemicals, such as metals, air pollutants, bisphenol A, and melamine, also play significant roles in CKD development and progression (4–6). Metals, whether essential or non-essential, are commonly found in water, soil, and air, with human exposure increasing due to industrialization. Cadmium(Cd) exposure, in particular, has been associated with severe kidney damage through the downregulation of specific microRNAs, thereby promoting apoptosis induced by cadmium and other heavy metals (7). Studies in Bangladesh have reported higher levels of lead (Pb), cadmium and chromium (Cr) in the urine of CKD patients compared to controls, suggesting a potential link between heavy metal exposure and CKD (8). However, existing research often fails to address combined exposure to multiple metals, overlooks covariance between them, and does not clarify the correlation between metal exposure and renal function parameters. The investigation into whether a linear or nonlinear dose-effect relationship exists between metal exposure and CKD progression remains an urgent matter for further exploration.

The objectives of this study are as follows: (1) Investigate the predictors of CKD and metal mixtures, both overall and across different subgroups. Analyze the variations in metal indicators based on different stages of CKD. (2) Compare the performance of various machine learning techniques in predicting metal features for CKD. (3) Evaluate the overall effect, interactions and non-linear relationships of metal mixtures on CKD. (4) Analyze the moderating effect of different metals on Creatine and Cystatin C, which influence the development of CKD.

## Patients and methods

### Study population and validation process

This cross-sectional study is based on data obtained from the Health and Examination Survey of Patients with CKD in Shenyang City, Liaoning Province, China. Conducted between November 2021 and August 2022, the survey comprehensively collected demographic and health data from 100 medical examiners and 443 CKD patients at the medical center of First Hospital Affiliated to China Medical University. The sample size was determined using equation recommended by Adhikari et al. (9), incorporating a significance level (α) of 0.05, an expected presenteeism rate (p) of 0.5, and a permissible error (δ) of 0.15p. This calculation yielded an estimated sample size of 267.


$$N=1.5\times \frac{z_{1-\alpha /2}^{2}}{\delta^{2}}\times p\times (1-p)$$

The inclusion criteria for study participation in the study were as follows: subjects with good compliance, capable of cooperating with investigators to complete the study and no history of infectious diseases, Parkinson’s disease, Alzheimer’s disease, etc. within the last 3 months. The exclusion criteria included: (1) subjects with abnormal results in routine laboratory tests; (2) patients currently diagnosed with diabetes mellitus, systemic lupus erythematosus, chronic hepatitis B with urinary tract infections, severe cardiopulmonary and hepatic diseases or other vital organ dysfunctions; and (3) patients currently taking traditional Chinese medicines, psychotropic medicines, non-steroidal anti-inflammatory medicines, glucocorticosteroids, or any other medications that could potentially affect renal function indicators.

The studies involving human participants were reviewed and approved by Ethics Committee of the First Hospital of China Medical University (ID: AF-SOP-07-1.1-01, Number: 2020-238.2). The patients/participants provided their written informed consent to participate in this study.

### Sociodemographic and disease characteristics

After reviewing the consultation platform and inpatient information system of the First Affiliated Hospital of China Medical University, we gathered data on study participants, including age, gender, and results from clinical examinations such as urea, creatinine, estimated glomerular filtration rate (eGFR), cystatin C. Continuous variables were described using mean (standard deviation) for symmetric distributions, or median (interquartile range (IQR)) for asymmetric distributions.

### Measurement of metal concentrations in serum

The remaining blood samples obtained after venous blood collection from the study subjects were centrifuged at 400 g/min for 5 minutes. Following centrifugation, the supernatant was carefully collected using a pipette and transferred to clean polypropylene tubes. Subsequently, the processed blood samples were stored at -20°C in a refrigerator before being uniformly transported to a deep-freeze refrigerator at -80°C in China Medical University for long-term storage. Special attention was given to maintaining low temperatures during transportation. For the determination of metal concentrations in the serum, an Agilent 7700x inductively coupled plasma mass spectrometer (ICP-MS, Agilent, USA) was utilized. This analysis covered 30 metals, including lithium (Li), beryllium (Be), boron (B), sodium (Na), magnesium (Mg), aluminum (Al), potassium (K), calcium (Ca), vanadium (V), Cr, manganese (Mn), iron (Fe), cobalt (Co), nickel (Ni), copper (Cu), zinc (Zn), gallium (Ga), arsenic (As), selenium (Se), rubidium (Rb), strontium (Sr), molybdenum (Mo), silver (Ag), Cd, antimony (Sb), cesium (Cs), barium (Ba), thallium (TI), Pb, and uranium (U). The procedure involved several steps: (1) Thawing the frozen serum samples overnight at 4°C, ensuring thorough mixing after thawing. (2) Pipetting 0.8 ml of 65% nitric acid and 0.2 ml of 30% hydrogen peroxide(CIAC, China) into 15 mL polypropylene tubes, then accurately measuring 0.25 mL of serum samples into the same tubes. The tubes were then placed uncapped on an electric heating plate to undergo a reaction at 90°C for 1 hour, followed by warming up the heating plate(Shenglan, China) to 110°C for acid expulsion over a 4-hour period. After cooling to room temperature, all samples were removed from the heating plate. (3) Diluting the solution to 10 mL with ultrapure water, capping the tube and mixing it upside down several times. Subsequently, 2-4 mL of the solution was filtered into a sample tube for sampling by ICP-MS. (4) Metal concentrations in samples below the Limit of Detection (LOD) were considered non-detected. These values were included in the analysis by replacing them with LOD/2, following the method established by the Experimental Laboratory Center and existing literature.

### Variable screening and correlation analysis

For this study, multiple imputation was conducted to fill in missing values, using a parameter m=5 and predictive mean matching (pmm) method. Factors influencing CKD, abnormal Creatine and Cystatin C levels were analyzed separately through univariate and multiple analyses. Additionally, differences in metal levels among CKD patients at various stages were explored. In univariate analyses, ANOVA and parametric tests were applied, while the LSD-t test or non-parametric tests were utilized for two-way comparisons. Multiple regression analysis involved two methods: lasso regression to examine the association between metal elements and CKD, and logistic regression to explore the relationship between metal elements and abnormal Creatinine and Cystatin C levels. In both multiple regression analyses, significant clinical and personal characteristic factors identified in univariate analysis were included as covariates to control the model.

### Machine learning training process

In this study, R software (version 4.1.3) was utilized. The input parameters consisted of metals and other factors associated with CKD disease, Creatine and Cystatin C abnormalities in the multiple regression models. To ensure consistent training, each model was trained on 75% of the training sample. A common 25% holdout set was retained for all models and used to generate statistics for result comparisons.

For predicting CKD problems, two alogorithms, namely Gaussian Process Regression (GPR) and Support Vector Machine (SVM), were employed. Each algorithm employed four kernel functions. After training, the performance of these algorithms was evaluated using various metrics of interest. Accuracy, AUC (internal validation), and F1 score were used to compare the performance of the classification algorithms. The algorithms exhibiting the best performance were chosen for external validation.

### Bayesian kernel machine regression (BKMR) model

We employed the BKMR model, a non-parametric Bayesian variable selection framework, to assess the combined effect of metals on CKD, Creatine and Cystatin C abnormalities. BKMR integrates Bayesian and statistical learning methods to iteratively regress an exposure–response function using a Gaussian kernel function. Given the high correlation among the metals in our analysis, we utilized a variable selection method with 20,000 iterations via a Markov chain Monte Carlo algorithm (10). Based on multiple regression techniques (logistic and lasso regression), we categorized certain metals into groups related to CKD disease, Creatine and Cystatin C abnormalities.


$$Y_{i}=h(z_{i1},\ldots ,z_{iM})+\beta^{T}X_{i}+e_{i}$$

Here *h()* was the exposure–response function, which accounts for nonlinearity and/or interaction among the mixture components, *Z_i_* denotes metals, while *X_i_* and *β* represent covariates and their coefficients, respectively. Covariates were selected through Spearman’s correlation analysis, prioritizing those with higher coefficients. Typically, a PIP threshold of 0.5 is employed to determine their importance (11).

### Analysis of moderating effects

In this study, Hayes process (v.3.5) within SPSS 21.0 (SPSS Inc., Chicago, IL, USA) was employed to validate the mediation hypotheses regarding a recent study. To assess the mediation effect of CKD diseases, Creatine and Cystatin C abnormalities on the predicted variables, a Hayes process moderation analysis was conducted, with 5000 bootstrapping-based resamples chosen (12). The mediating factors selected were the metal factors exhibiting significance and substantial effect values in the multiple analyses.

## Results

### Comparison of general and clinical characteristics within groups

**Supplementary Table 1** demonstrates that there were statistically significant differences in the general and clinical characteristics between the case and control groups, particularly in age, gender, Na ion, K ion, serum albumin, and platelet count. Notably, the eGFR in the case group was significantly lower compared to the control group, while leukocyte count and granulocyte ratio were notably higher. Similarly, significant differences in general characteristics were observed between abnormal and normal creatinine groups, with exceptions noted for chloride ion. Variables such as eGFR, Na ion, bicarbonate ion, Ca ion, serum albumin, lymphocyte ratio, hemoglobin concentration, and platelet count were lower in the abnormal creatinine group compared to the normal creatinine group. Conversely, age, K ion, anion gap, phosphorus ion, Mg ion, leukocyte count, and granulocyte ratio were higher in the abnormal creatine group. Similar variations were observed between the normal and abnormal Cystatin C groups.

### Comparison of serum concentrations of metallic elements between different subgroups

**Table 1** illustrates the variations in metal concentrations across different groups. With the exception of Li, Al, V, Co, Zn, Sb, TI, and Pb, all other metals exhibited significant differences between the case and control groups. Specifically, concentrations of Be, B, Na, K, Cr, As, Rb, Mo, Cd, Cs, and U were notably higher in the case group compared to the control group (P<0.05). Moreover, in the abnormal creatinine group, concentrations of Mg, Ca, Fe, Cu, Se and Pb, were significantly lower compared to the normal creatinine group (P<0.05). Similarly, in the cystatin C abnormal group, concentrations of Mg, Ca, Mn, Fe, Cu, Se and Pb were markedly lower than those in the cystatin C normal group.

**Table 1:** Description of CKD, creatinine and cystatin C abnormalities with metals.

Metallic Element	CKD (Median (IQR))		P(CKD)	Creatine (Median (IQR))		P(Creatine)	CystatinC (Median (IQR))		P(CystatinC)
	Case	Control		Abnormal	Normal		Abnormal	Normal	
Li(μg/L)	15.72(0,59.43)	23.07(1.06,51.52)	0.289	17.64(0,60.32)	16.48(0,53.68)	0.758	16.64(0,61.24)	18.39(0,52.65)	0.596
B(μg/L)	0.97(0,2.88)	0.03(0,2.44)	0.021	0.95(0,2.69)	0.22(0,3.04)	0.187	0.99(0,2.8)	0.14(0,2.65)	0.045
B(μg/L)	103.35(24.23,174.85)	0(0,0)	<0.001	112.11(26.31,186.33)	0(0,67.46)	<0.001	110.15(32.09,185.29)	0(0,49.22)	<0.001
Na(μg/L)	6737834.75(6382079.43,7037459.44)	3207461.19(3097188.28,3520188.66)	<0.001	6671799.31(6288770.52,7005872.31)	6313184.58(3241400.2,6896912.15)	<0.001	6725231(6358463.92,7029559.91)	3716379.17(3166540.65,6729512.78)	<0.001
Mg(μg/L)	13108.06(10219.86,19225.54)	38918.49(28942.95,51146.2)	<0.001	13910.46(10639.6,21449.06)	18381.57(11357.94,36250.18)	<0.001	13513.5(10516.32,20611.8)	24765.02(11891.26,40736.03)	<0.001
Al(μg/L)	2331.03(0,6484.49)	1878.82(885.87,2588.08)	0.057	2178.01(0,6202.72)	2009.56(274.38,5149.17)	0.787	2365.3(0,6414.44)	1916.36(429.77,3999.72)	0.197
K(μg/L)	729880.74(641934,817517.94)	160351.52(150978.58,175536.14)	<0.001	721782.7(619240.7,819146.9)	587167.47(163921.42,755359.58)	<0.001	730161.02(636411.31,821191.03)	184895.82(158421.36,710716.03)	<0.001
Ca(μg/L)	11378.59(7195.44,15920.71)	25620.31(20288.31,33822.64)	<0.001	13101.26(8779.52,18242.53)	13372.63(6003.23,24498.74)	0.0452	12113.8(8470.12,16761.39)	17570.8(6833.32,27426.66)	<0.001
V(μg/L)	0(0,0.55)	0.04(0,0.79)	0.072	0(0,0.61)	0(0,0.55)	0.457	0(0,0.57)	0(0,0.59)	0.857
Cr(μg/L)	5.28(0,22.19)	0(0,7.71)	0.001	6.07(0,23.14)	0(0,12.98)	0.001	8.4(0,23.63)	0(0,9.45)	<0.001
Mn(μg/L)	0(0,11.93)	18.63(8.55,37.21)	<0.001	0(0,16.82)	0.04(0,20.06)	0.078	0(0,13.47)	8.19(0,25.18)	<0.001
Fe(μg/L)	984.55(657.95,1534.15)	1902.78(1378.06,2546.68)	<0.001	1035.41(674.55,1705.04)	1331.4(825.37,2035.75)	0.001	985.11(663.24,1584.26)	1416.71(931.58,2219.08)	<0.001
Co(μg/L)	0.22(0,0.64)	0.21(0,0.61)	0.12	0.23(0,0.69)	0.17(0,0.6)	0.061	0.23(0,0.69)	0.16(0,0.57)	0.01
Ni(μg/L)	0.77(0,4.94)	2.65(0.6,6.51)	<0.001	1.15(0,5.08)	1.14(0,5.9)	0.354	1.01(0,4.79)	1.65(0,6.47)	0.052
Cu(μg/L)	622.32(481.49,777.51)	957.14(802.06,1142.33)	<0.001	635.15(483.42,785.37)	788.86(577.78,980.35)	<0.001	633.13(481.09,793.46)	792.39(608.13,992.37)	<0.001
Zn(μg/L)	878.03(82.99,2776.85)	1006.85(136.87,1526.86)	0.685	889.79(104,2161.02)	963.4(104.56,1690.28)	0.625	877.44(83.79,2398.25)	978.41(122.12,1687.15)	0.897
Ga(μg/L)	0(0,0)	0(0,0.02)	<0.001	0(0,0)	0(0,0)	0.001	0(0,0)	0(0,0)	<0.001
As(μg/L)	0.95(0,2.21)	0.14(0,1.31)	<0.001	1.24(0.2,2.4)	0.02(0,1.08)	<0.001	1.18(0.19,2.4)	0(0,1.1)	<0.001
Se(μg/L)	50.08(39.21,62.75)	92.77(85.69,105)	<0.001	50.02(39.64,63.94)	72.72(50.78,89.19)	<0.001	49.32(38.59,62.05)	82.3(56.48,94.77)	<0.001
Rb(μg/L)	153.92(134.99,176.36)	147.38(130.17,161.87)	0.027	154.93(135.75,177.85)	147.54(131.24,165.08)	0.003	153.89(134.84,177.88)	148.49(133.71,165.12)	0.027
Sr(μg/L)	54.98(28.38,80.54)	57.81(26.95,103.86)	0.023	63.73(40.08,86.43)	33.28(11.21,61.67)	<0.001	61.23(38.16,84.29)	35.13(12.84,84.48)	<0.001
Mo(μg/L)	4.91(0,15.53)	0(0,0)	<0.001	4.81(0,11.73)	0(0,6.72)	0.004	4.95(0,13.99)	0(0,4.55)	<0.001
Ag(μg/L)	0(0,0.09)	0(0,0)	<0.001	0(0,0.07)	0(0,0)	0.004	0(0,0.1)	0(0,0)	<0.001
Cd(μg/L)	0.06(0,0.15)	0.02(0,0.1)	0.001	0.06(0,0.13)	0.05(0,0.16)	0.655	0.06(0,0.14)	0.04(0,0.12)	0.072
Sb(μg/L)	11.56(0,31.34)	9.27(5.6,14.92)	0.52	10.87(0,28.94)	10.05(1.99,26.73)	0.324	11.66(0,31.18)	9.27(1.63,18.63)	0.297
Cs(μg/L)	0.45(0.25,0.72)	0.15(0,0.49)	<0.001	0.47(0.29,0.73)	0.24(0,0.51)	<0.001	0.47(0.29,0.73)	0.21(0,0.52)	<0.001
Ba(μg/L)	20.43(6.86,33.74)	26.56(12.26,45.96)	0.013	20.48(8.53,34.2)	21.55(8.62,40.13)	0.404	20.4(8.65,33.79)	22.88(8.56,39.97)	0.337
Tl(μg/L)	0(0,0)	0(0,0)	0.071	0(0,0)	0(0,0)	0.606	0(0,0)	0(0,0)	0.817
Pb(μg/L)	0(0,8.29)	1.06(0,7.7)	0.104	0(0,7.52)	1.93(0,8.94)	0.001	0(0,7.43)	1.68(0,8.57)	0.004
U(μg/L)	0.01(0,0.1)	0(0,0)	<0.001	0.01(0,0.1)	0(0,0.07)	0.002	0.02(0,0.11)	0(0,0.04)	<0.001

In the CKD population, a 5-fold cross-validation approach was employed to select stable models with minimal error fluctuations for analysis. Parameter λ values of 0.016 for Model 1 and 0.017 for Model 2 were utilized. The results, presented in **Table 2** and **Supplementary Figure 1**. indicated that after adjusting for covariates such as sex, age, ethnicity, marital status, occupation, and region of residence, Mg, Ca, Cu, and Se were negatively associated with CKD (coefficient<0), while Na, K, and Ag showed positive associations with CKD (coefficient >0).

**Table 2:** Multivariate analysis of the association of CKD and concentration changes in serum metals.

Parameters (μg/L)	Model 1	Model 2
	Coefficients	Coefficients
B	4.03E-05	–
Na	7.89E-08	7.88E-08
Mg	-9.51E-07	-6.73E-07
K	3.17E-07	2.22E-07
Ca	-4.59E-06	-5.82E-06
Cu	-9.26E-05	-1.01E-04
Se	-3.72E-03	-3.38E-03
Rb	-2.09E-04	–
Ag	3.27E-03	1.62E-03
Model parameters	λ=0.016 CV=5	λ=0.017 CV=5

Model 1: adjusted for metals for univariate analyzes.

Model 2: model 1 plus sociodemographic and general blood indicators for univariate analyzes.

### Variability in metal levels across CKD stages

Regarding the variability in metal levels across different stages of CKD, significant differences were observed among the stages for twenty metal indicators (**Supplementary Table 2**). Except for Na, Cd, and Sb, which mainly differed between the early and late stages, all other metallic elements exhibited variations across different stages. Metals that significantly differed between each stage were present throughout all stages. Further details can be found in **Supplementary Table 2**.

### Multifactorial regression

**Supplementary Table 3** presents the results of multifactorial regression analysis for the creatinine abnormality group. In both Model 1 and Model 2, Se demonstrated a negative effect, while As exhibited a negative effect only in Model 2. Additionally, Fe and Rb showed positive effects in Model 2. **Supplementary Table 4** displays the aggregated and stratified regression results for individuals with abnormal cystatin C levels. Both models indicated that K and Pb had positive effects, while Mo showed a positive effect only in Model 2. Se had a negative effect in both Model 1 and Model 2 (**Supplementary Tables 3**, **4**).

### Algorithm selection and prediction effect

**Figure 1:**
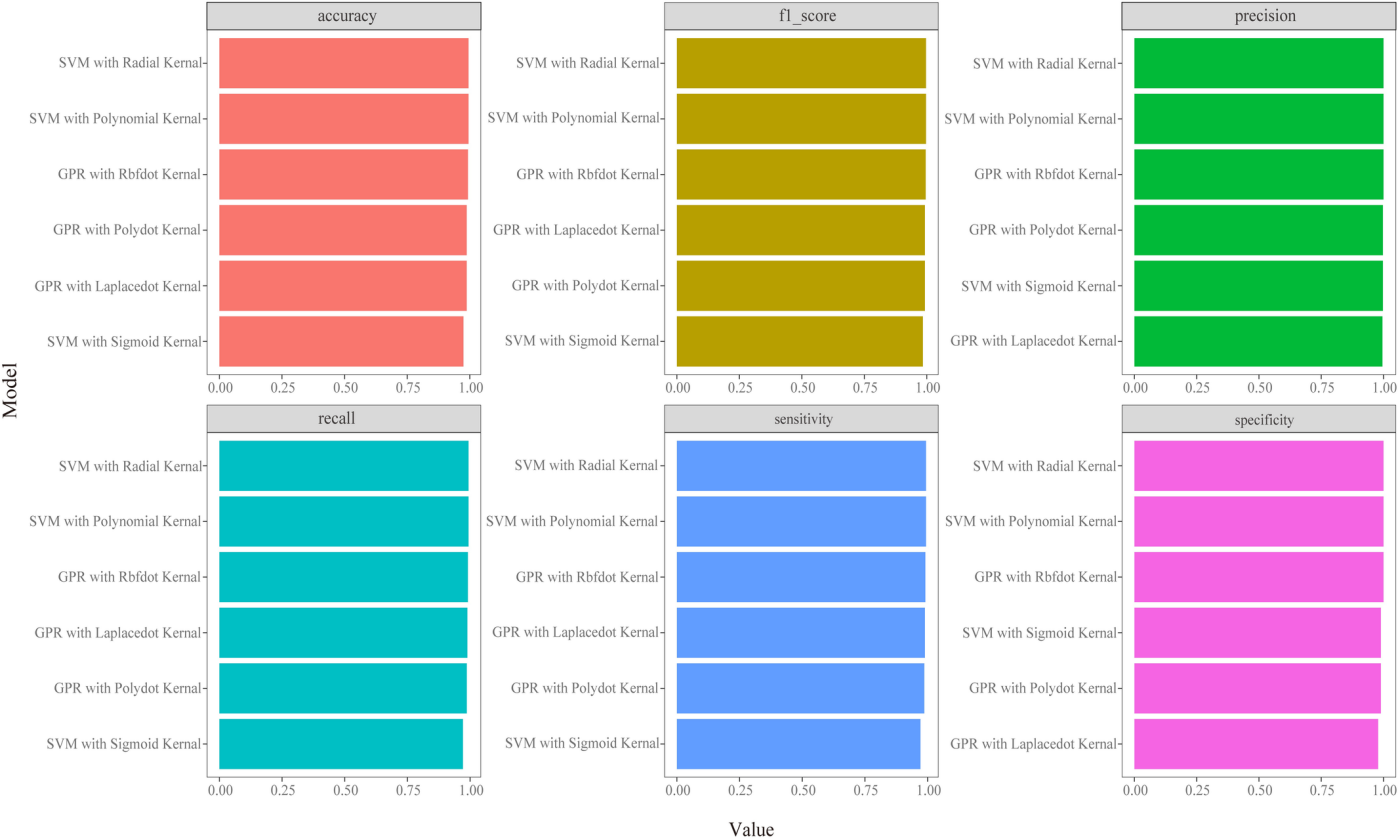
Classifiers’ performance metrics comparison in CKD disease model of different machine learning algorithms. Each model’s accuracy was checked by an accuracy, precision, recall, F1 score, sensitivity and specificity.

To select an appropriate algorithm for prediction, we conducted multifactorial regression analysis and summarized the performance of six maximum likelihood algorithms in estimating indicators such as laboratory tests and metal concentrations, as well as predicting the onset of CKD and abnormalities in creatinine and cystatin C levels (**Figure 1**; **Supplementary Figures 2**-**6**). Based on the results, SVM-Radial and GPR-Laplacedot demonstrated the best performance. Subsequently, we trained and validated both models, and the results in **Supplementary Table 5** indicated a good fit between the training and test sets (R^2^∈(0.7-0.9)).

### BKMR analyses

**Figure 2:**
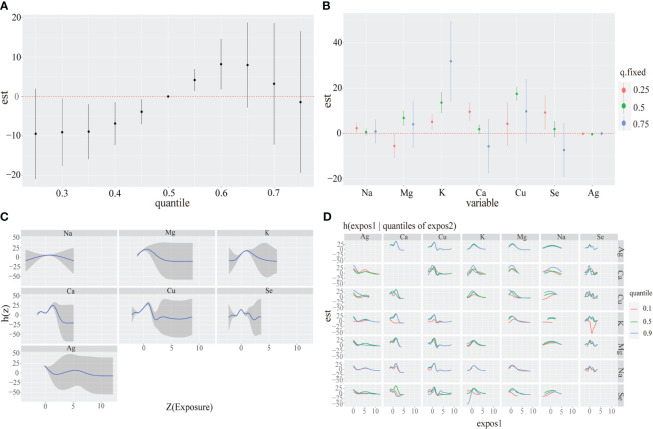
Associations between serum metal mixtures and CKD disease among the study population by BKMR model. Model adjust for Sex, Age and other laboratory indicators by using Spearman correlation. **(A)** The cumulative effect of the serum metal mixtures (estimates and 95% credible intervals). Metal mixtures are at a particular percentile (X-axis) compared to when exposures are all at 50th percentile. **(B)** The single-exposure effect (estimates and 95% credible intervals). **(C)** Univariate exposure-response functions and 95% confidence bands for each serum metal with the other mixtures fixed at the median. **(D)** Multiple exposure-response functions for: the other metal when one metal fixed at either the 25th, 50th, or 75th percentile and the test of metal mixtures is fixed at the median.

We presented the visualization of the BKMR model with **Supplementary Table 6** summarizing the probabilities of inclusion (PIPs), which were relatively high in both models. Initially, we observed the cumulative effect of metal mixtures on CKD disease showing a trend of increasing and then decreasing total effect with exposure time. Particularly, the overall effect was statistically significant when all metabolites were between the 30th and 60th percentile compared to when all metal mixtures were at their median values (**Figure 2A**). Next, we examined the individual effects of metal mixtures by analyzing the change in CKD disease associated with variations in single metal mixtures from their 25^th^ percentile to 75^th^ percentile while keeping other metals at specific thresholds (25th, 50th, or 75th percentiles). Serum K and Ca exhibited significant positive effects. For instance, an increase in serum K concentration from the 25^th^ to the 75^th^ percentile was associated with a significant rise in CKD disease of 5.15(1.77,8.53), 13.62(8.91,18.33) and 31.81(14.03,49.58) when other metals are fixed at the 25^th^, 50^th^ and 75^th^ percentiles, respectively. In the same way, serum Ca concentration from 25^th^ to 50^th^ is associated with a significant decrease in CKD disease of 9.60(5.58,13.62), 1.88(-0.19,3.95) and -5.65(-17.56,6.27) (**Figure 2B**). To explore potential nonlinearity in the exposure-response function, we analyzed the univariate relationship between each metal and CKD disease, with other metals fixed at the 50th percentile. The plot suggested nonlinear effects of serum Na, Mg and K, showing an inverted U-shaped relationship with CKD disease (**Figure 2C**). Further investigating the relationship between serum metal mixtures, we plotted bivariate cross-sections of exposure-response function. **Figure 2D** illustrates differences in CKD disease as a function of Se, varying Se concentrations from the 25^th^ to 50^th^ and to 75^th^ percentiles while fixing all other metal mixtures at their medians.

Although no statistically significant difference was found in the abnormal Creatine model, there was an increasing trend (**Supplementary Figure 7A**). We observed that serum Ca and Rb had a significant positive effect, while the opposite was true for Se. Specifically, a change in serum Ca and Rb concentration from the 25^th^ to the 75^th^ percentile was associated with a significant increase in abnormal Creatine by 0.58(-0.17,0.99) and 0.86(-0.22,1.50), respectively, whereas a change in serum Se concentration showed a significant decrease by 0.30(-0.04,0.57) and 0.37(-0.14,0.59) (**Supplementary Figure 7B**). The plot suggested linear effects of serum Na, K and Se, with increasing levels of serum K and Se correlating with a significant decrease in abnormal Creatine (**Supplementary Figure 7C**). Evidence of interaction between serum Ca and Se was demonstrated by parallel exposure-response relationships (**Supplementary Figure 7D**).

We observed that the total effect of abnormal Cystatin C tended to decrease with increasing exposure time. Particularly, the overall effect was statistically significant when all metabolites were between the 45^th^ and 65^th^ percentile compared to when all metal mixtures were at their median values (**Supplementary Figure 8A**). Serum Se and Mo exhibited a significant negative effect, whereas K showed the opposite trend. Specifically, a change in serum Se and Mo concentration from the 25^th^ to the 75^th^ percentile was associated with a significant decrease in the abnormal Cystatin C by -1.12(-1.57,-0.66), -1.14(-1.48,-0.79) and -1.73(-2.38,-1.08), -0.25(-0.74,0.24), -0.75(-1.15,-0.34) and -1.02(-1.63,-0.40), respectively. Conversely, a change in serum K concentration from the 25^th^ to the 75^th^ percentile was associated with a significant increase in abnormal Cystatin C by 0.28(-0.05,0.61), 0.34(-0.06,0.61) and 0.39(-0.01,0.79) (**Supplementary Figure 8B**). The plot suggested linear effects of serum K, where increasing serum K levels correlate with a significant increase in abnormal Creatine (**Supplementary Figure 8C**). Evidence of interaction between serum K and Mo was shown by parallel exposure-response relationships (**Supplementary Figure 8D**).

### Analysis of the mediating effect

**Figure 3:**
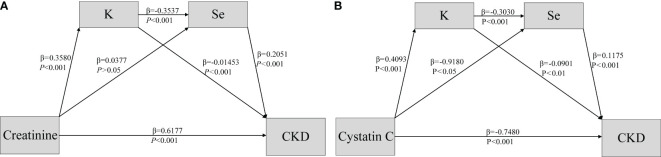
Association of Creatine & Cystatin C **(A, B)** and CKD disease (Y) mediated by potassium (M1) and selenium (M2).

As shown in **Table 3**, after screening for various metallic elements, the bootstrap 95% confidence intervals (CI) for the total indirect effect of K and Se did not contain 0, indicating a significant mediating role of K and Se in CKD. Moreover, all four pathways contributed to mediating effects in both models, with showing significant indirect effects. The pathways of creatinine and cystatin C on CKD are illustrated in **Figures 3A, B**.

**Table 3:** Proportion of the mediating effect.

Model	Effect	Path relationships	Coefficient	Boot[LLCI,ULCI]
1	Direct effect	Creatine→CKD	-0.0835	**[-0.0917,-0.0753]**
	Mediating effect	Creatine→K→CKD	-0.007	**[-0.0106,-0.0041]**
		Creatine→Se→CKD	0.001	**[-0.0106,-0.0041]**
		Creatine→K→Se→CKD	-0.0035	**[-0.0059,-0.0019]**
	Total effect		-0.0095	**[-0.0135,-0.0059]**
2	Direct effect	Cystatin C→CKD	-15.4473	**[-16.5321,-14.3626]**
	Mediating effect	Cystatin C→K→CKD	-0.7613	**[-1.2052,-0.3313]**
		Cystatin C→Se→CKD	-0.2228	**[-0.4757,-0.0009]**
		Cystatin C→K→Se→CKD	-0.3007	**[-0.5554,-0.1393]**
	Total effect		-1.2847	**[-1.7778,-0.8125]**

Model 1:Creatine→K→Se→CKD.

Model 2:Cystatin C→K→Se→CKD.

Bold font indicates statistical significance at the 0.05 level.

## Discussion

In this study we employed multifactorial regression to investigate the association between serum metals and CKD. Interestingly, our findings diverged significantly from previous studies (13, 14), which could be attributed to geographic variations, differences in dietary habits, lifestyles, and environmental conditions (15) across different regions. Through LASSO regression analysis, we identified a positive association between Na and CKD, even after adjusting for covariates such as general characteristics and routine blood markers. This aligns with the results of a retrospective cohort study (16), possibly because excessive Na intake can elevate blood pressure, a known risk factor for CKD (17). However, it has also been observed that both low and high concentrations of Na ion are associated with increased mortality risk in CKD patients (18). The mechanisms underlying how Na affects renal function remain inadequately understood and warrant further investigation in future studies. Studies have indicated an independent and statistically significant U-shaped association between serum K levels and mortality in CKD patients (19). In contrast, our study only found a positive correlation between serum K and CKD, which may be attributed to disparities in study populations, regions, and statistical methods. Prior research has consistently shown that serum Mg concentration is lower in CKD patients compared to controls and is negatively correlated with CKD development, consistent with the findings of Vermeulen and Vervloet (20). As an essential trace element, the positive influence of Se supplementation on CKD pathogenesis reaffirms our results of this study (21–23). However, the association between Se and CKD remains contentious, as some studies have suggested that Se supplementation may be detrimental to CKD patients (24, 25). Our study observed a significant correlation between plasma Rb and rapid decline in renal function. However, limited data exists regarding the role of Rb in renal dysfunction (26, 27). Usuda et al. proposed that Rb can exhibit diverse biological effects depending on its anionic valence state (28).

Traditional logistic regression analysis revealed positive correlations between Na and K levels with CKD, while Se showed a negative correlation. Utilizing the BKMR-CKD model, significant positive cumulative effects were observed for metals such as K, Ca concentration in serum at 50-60^th^ percentile. Conversely, the BKMR-Cystatin C model indicated a positive exposure effect of serum K at 40-50^th^ percentile for metal mixtures, along with a negative exposure effect of Se, Mo at 50-70^th^ percentile. Disruptions in high levels of K ions distribution due to tissue damage, metabolic acidosis with normal anion gap, and increased tension may adversely affect renal function and its complications (29). Regarding Ca’s effect, both positive and negative effects were noted, reflecting the importance of maintaining balance in CKD patients. Negative balance could heighten the risk of osteoporosis and fractures, while positive balance may increase the likelihood of vascular calcification and cardiovascular events (30).Se has been shown to modulate eGFR in patients with environmental toxicant-induced chronic renal failure, playing a crucial role in mitigating CKD. These findings offer potential insights into environmental exposures and the role of Creatine- and Cystatin C-mediated CKD progression. In the context of metal mixtures, one study revealed that high plasma Se concentrations interacted additively and multiplicatively with low erythrocyte Pb and Cd levels, significantly impacting CKD (31). The interaction of K and Se in CKD progression in this study remains unconfirmed, possibly stemming from oxidative stress and the elemental balance within serum metals.

This study employed various modeling methods, to effectively address different research objectives. Initially, traditional logistic regression was used to explore the association between serum metal levels and CKD, but the results were unsatisfactory. Therefore, regularization techniques such as lasso regression were utilized to overcome the limitations of the traditional approach. Through lasso regression, which aimed to investigate the link between serum metal levels and abnormal kidney indicators, numerous metal factors were selected, making it challenging to draw specific conclusions. Hence, for this study, employing the traditional logistic regression model for the indicators help reduce the interference from metal factors with lower weights. Subsequently, BKMR model analysis was conducted. After selecting appropriate metals via logistic and lasso regression, it delved deeper into the key metal factors influencing the indicators. Moreover, it conducted analyses on overall effect, dose-response relationships, and interactions of metal mixtures. This approach effectively addressed the limitations of traditional models, which often focused solely on singular effects of factors.

The current study has several strengths. Firstly, it includes multiple renal function indicators, providing a comprehensive understanding of the effects of metals on renal function. This holistic approach enhances the validity of the findings. Secondly, the study takes into account the interactions between metals, recognizing that other metals can either mask or exaggerate the true associations with renal impairment. This consideration lays a theoretical foundation for future research exploring and validating the effects of metal mixtures on CKD. Thirdly, a new flexible statistical method, the BKMR model is utilized to quantify and visualize the cumulative effects and dose-response relationships of serum metal mixtures in continuous variables. This approach reduces measurement bias and overcomes limitations associated with traditional analytical methods (32). However, this study also has some limitations. Firstly, it is a cross-sectional study, which only examines the correlation between metals and indicators of renal impairment without confirming a causal relationship between metals and CKD. Future longitudinal cohort studies and animal experiments are needed to establish causality. Additionally, the study acknowledges that dialysis patients with renal impairment may have altered clearance of metals, leading to elevated serum metal concentrations that may not reflect the actual situation. This may introduce errors in the results. Furthermore, confounding factors such as genetic variants, medication use, and other environmental pollutants in the residential area are potential sources of bias that cannot be ruled out. The study highlights the need for further longitudinal investigations to deeply explore the relationship between heavy metal concentrations and kidney function damage.

## Conclusion

We utilized multivariable logistic regression and BKMR models to assess the relationship between CKD and metal mixtures. The findings revealed positive associations between CKD and serum levels of K, Na and Ca, while Se and Mo exhibited negative associations. This highlights the importance of employing diverse methods to evaluate the health effects of metal mixtures. Additionally, our results suggest that BKMR model may serve as a useful tool for larger-scale mixture studies in the future. Machine learning techniques could be employed to validate the external validity of the identified metal factors.

## Data availability statement

The data are not publicly available due to restrictions that could compromise the privacy of research participants. Requests to access the datasets should be directed to kerk1982@163.com.

## Ethics statement

The studies involving humans were approved by The Ethics Committee of the First Hospital of China Medical University. The studies were conducted in accordance with the local legislation and institutional requirements. The participants provided their written informed consent to participate in this study. Written informed consent was obtained from the individual(s) for the publication of any potentially identifiable images or data included in this article.

## Author contributions

MW: Formal analysis, Investigation, Methodology, Software, Validation, Visualization, Writing – original draft, Writing – review & editing. WH: Formal analysis, Methodology, Software, Validation, Visualization, Writing – original draft, Writing – review & editing. RQ: Conceptualization, Formal analysis, Methodology, Software, Visualization, Writing – original draft, Writing – review & editing. GW: Formal analysis, Investigation, Methodology, Software, Writing – original draft, Writing – review & editing. DS: Formal analysis, Investigation, Methodology, Resources, Writing – original draft, Writing – review & editing. YG: Formal analysis, Investigation, Methodology, Resources, Software, Writing – original draft, Writing – review & editing. YD: Conceptualization, Data curation, Formal analysis, Funding acquisition, Investigation, Methodology, Resources, Validation, Writing – original draft, Writing – review & editing.
