# Supplementary material for: Comparative mathematical modeling of causal association between metal exposure and development of chronic kidney disease
Source: Front Endocrinol (Lausanne). 2024 May 1;15:1362085. doi: 10.3389/fendo.2024.1362085 (PMC11094205; doi:10.3389/fendo.2024.1362085)
Supplement: Supplementary file 1 [file DataSheet_1.docx]

**Supplementary materials**

**Contents**

**1.FIGURE S1** LASSO regression showed λ=0.016 and 0.017 when the error of the CKD model is minimized, and variables were selected for further multiple regression analysis without (a) or with adjustment (b).…………………………………………S1

**2.FIGURE S2** AUC-ROC plot of the CKD classifiers in different machine learning algorithms. TPR: True positive rate, FPR: false positive rate.………………………S2

**3.FIGURE S3** Classifiers' performance metrics comparison in creatinine abnormalities model of different machine learning algorithms. Each model's accuracy was checked by an accuracy, precision, recall, F1 score, sensitivity and specificity.......................S3

**4.****FIGURE S4** AUC-ROC plot of the creatinine abnormalities classifiers in different machine learning algorithms. TPR: True positive rate, FPR: false positive rate .…..S4

**5.FIGURE S5** Classifiers' performance metrics comparison in cystatin C abnormalities model of different machine learning algorithms. Each model's accuracy was checked by an accuracy, precision, recall, F1 score, sensitivity and specificity.…………..…S5

**6.FIGURE S6** AUC-ROC plot of the cystatin C abnormalities classifiers in different machine learning algorithms. TPR: True positive rate, FPR: false positive rate……S6

**7.FIGURE S7** Associations between blood metal mixtures and creatinine abnormalities among the study population by BKMR model………………………S7

**8.FIGURE S8** Associations between blood metal mixtures and cystatin C abnormalities among the study population by BKMR model.…………...…………S8

**9.****TABLE S1** Description of CKD, creatinine and cystatin C abnormalities with general and clinical data.…………….……………………………………...…………….…S9

**10.TABLE S2** Differences in metallic elements within different stages of chronic kidney disease (CKD)………………………………………………………………S10

**11.TABLE S3** Multivariate analysis of the association of creatinine abnormalities and concentration changes (95%CI) in blood metals.………………….………………...S11

**12.TABLE S4** Multivariate analysis of the association of cystatin C abnormalities and concentration changes (95%CI) in blood metals.………………………………..…. S12

**13.TABLE S5** Summary of the prediction results with different models including CKD disease, creatinine and cystatin C abnormalities...………………………………….S13

**14.TABLE S6** Posterior inclusion probabilities (PIPs) for metal mixtures into different groups models, using the Bayesian kernel machine regression (BKMR) model.…..S14


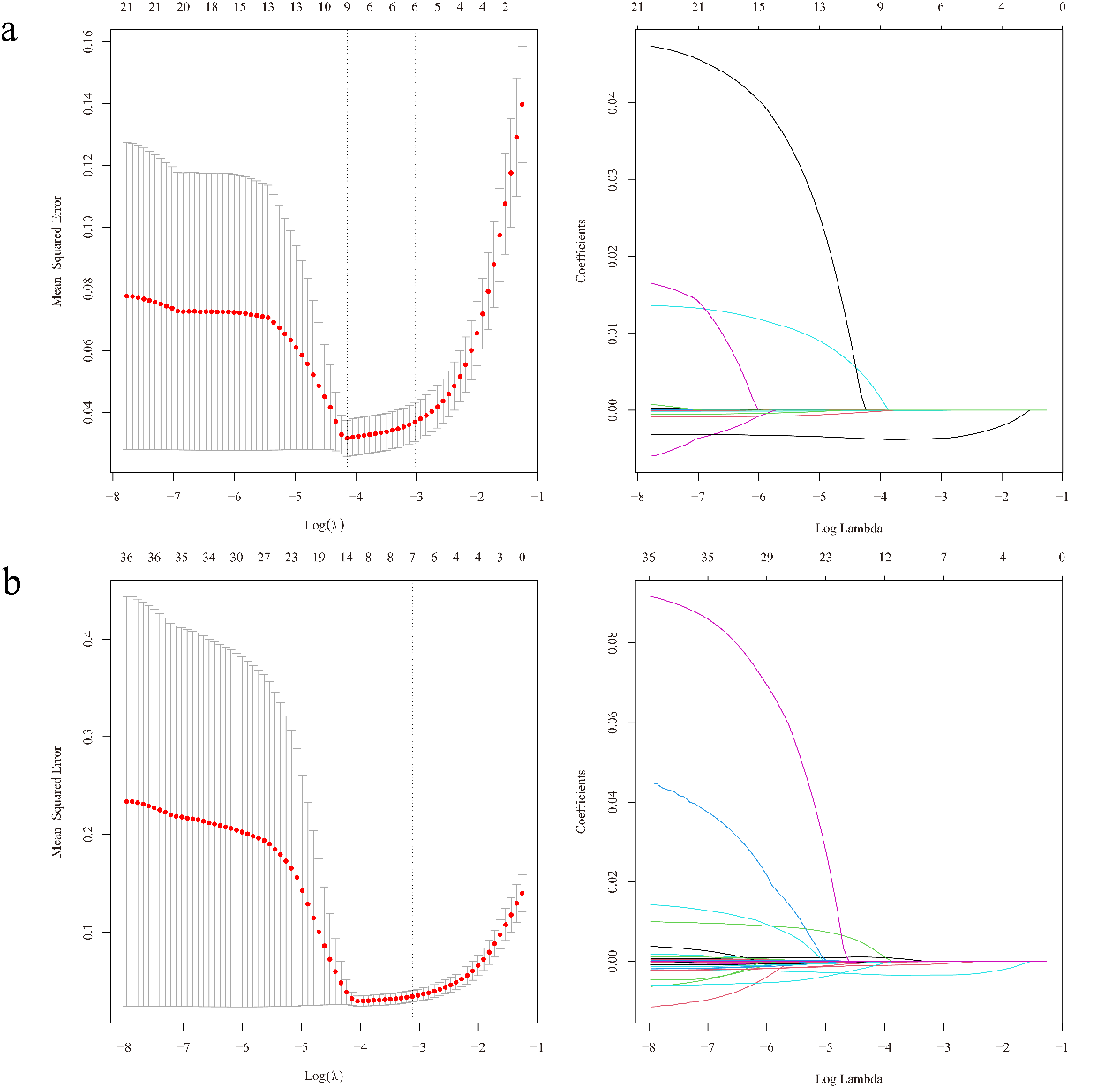
**FIGURE S1** LASSO regression showed λ=0.016 and 0.017 when the error of the CKD model is minimized, and variables were selected for further multiple regression analysis without(a) or with adjustment(b).


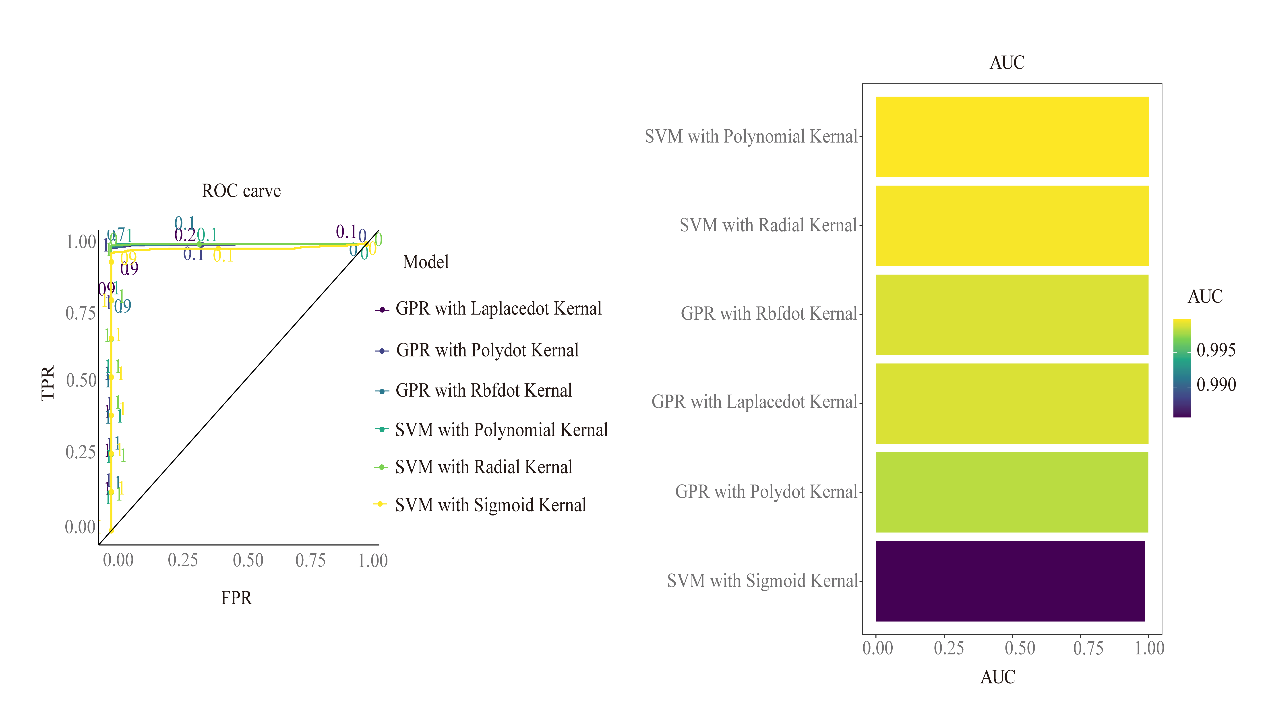
 **FIGURE S2** AUC-ROC plot of the CKD classifiers in different machine learning algorithms. TPR: True positive rate, FPR: false positive rate.


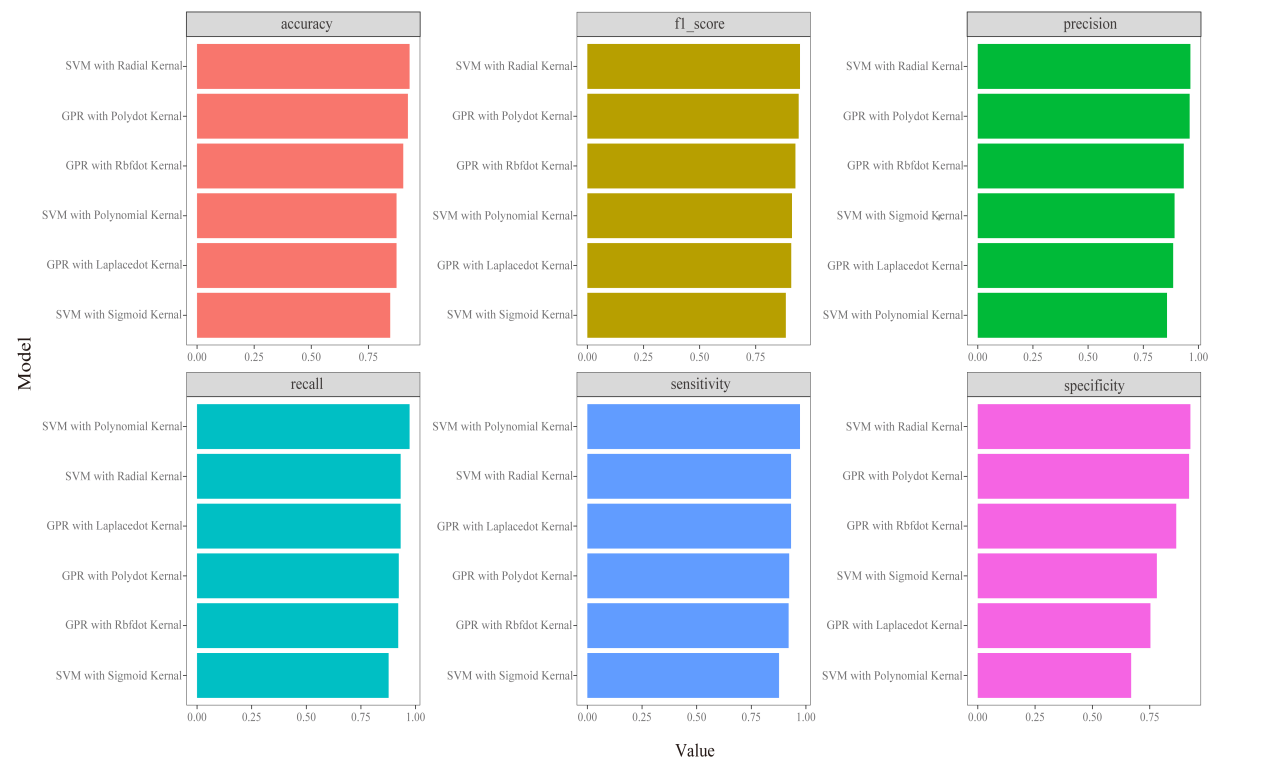
**FIGURE S3** Classifiers' performance metrics comparison in creatinine abnormalities model of different machine learning algorithms. Each model's accuracy was checked by an accuracy, precision, recall, F1 score, sensitivity and specificity.


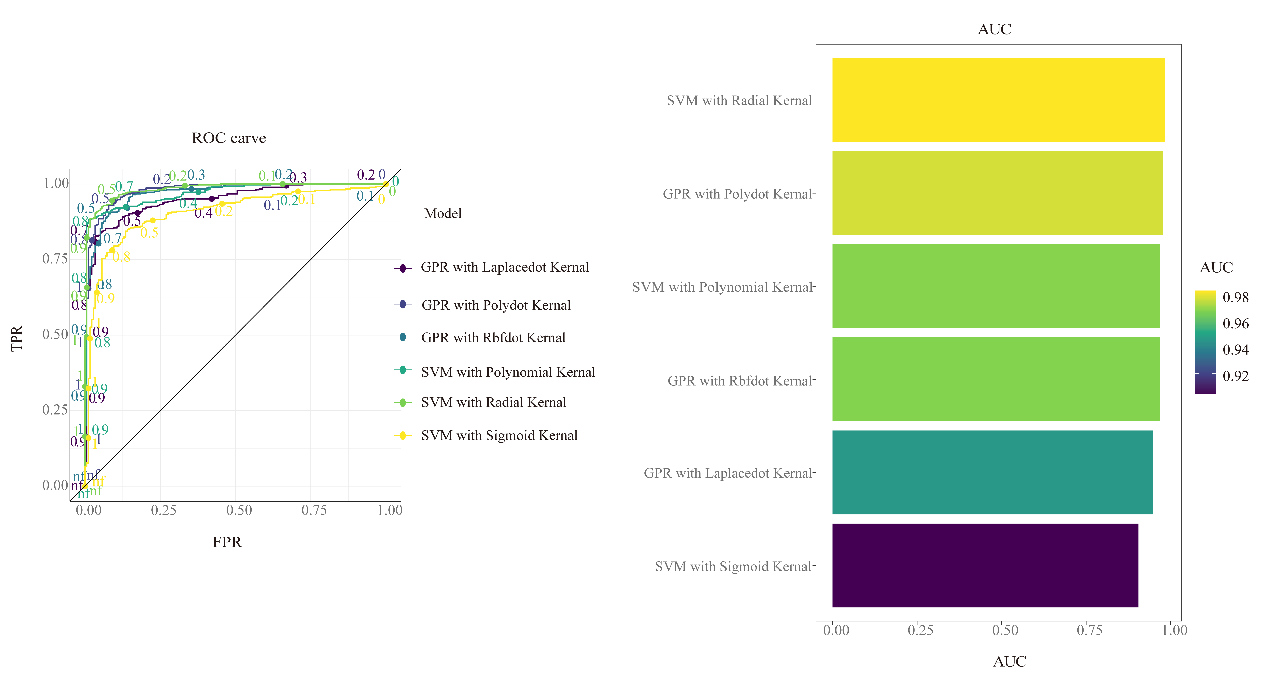


**FIGURE S4** AUC-ROC plot of the creatinine abnormalities classifiers in different machine learning algorithms. TPR: True positive rate, FPR: false positive rate.


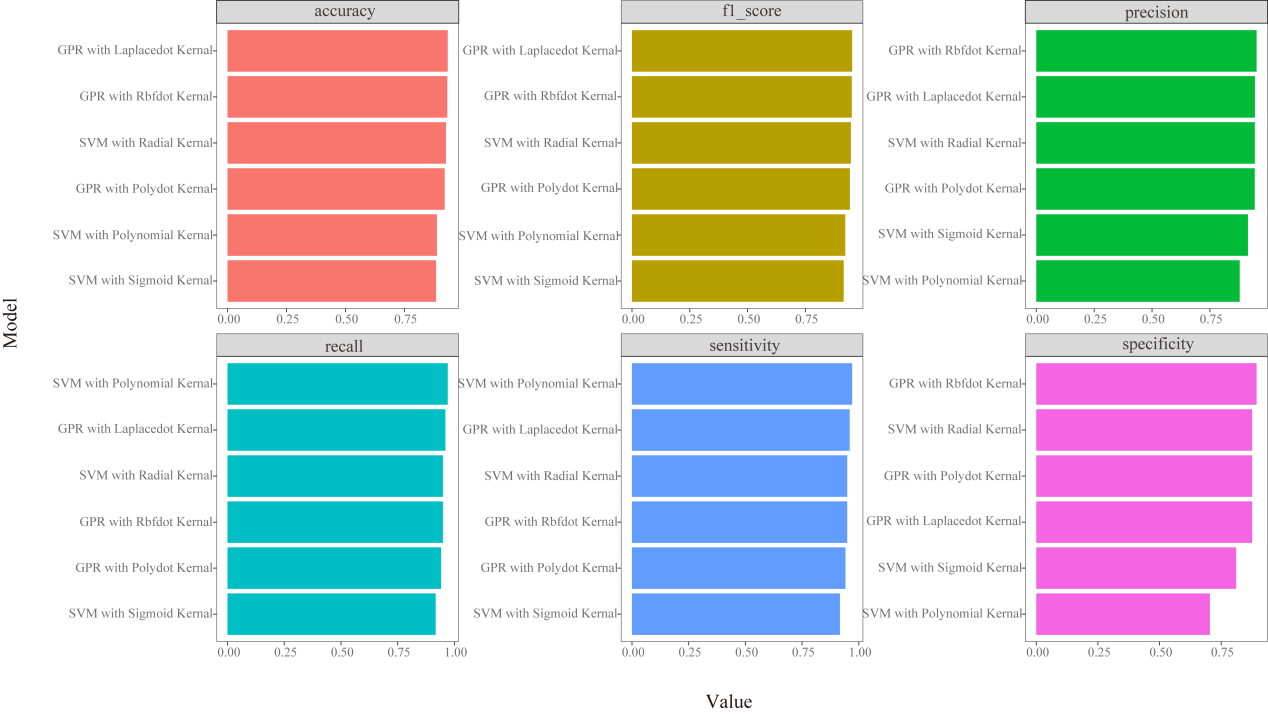
**FIGURE S5** Classifiers' performance metrics comparison in cystatin C abnormalities model of different machine learning algorithms. Each model's accuracy was checked by an accuracy, precision, recall, F1 score, sensitivity and specificity.


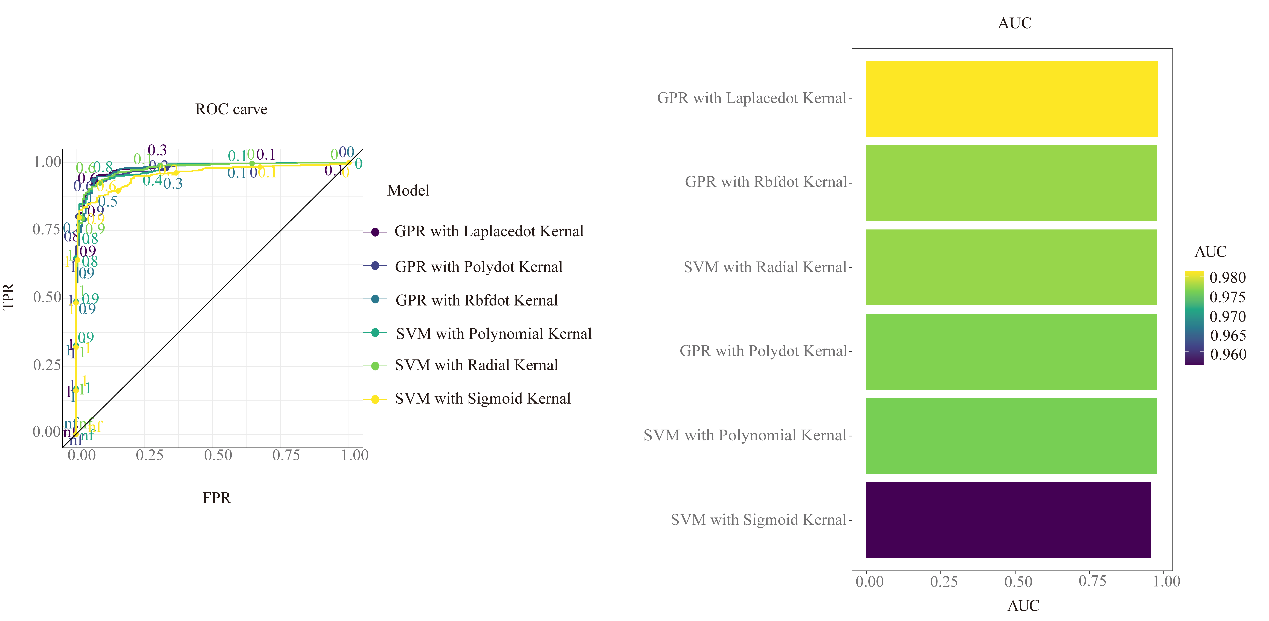


**FIGURE S6** AUC-ROC plot of the cystatin C abnormalities classifiers in different machine learning algorithms. TPR: True positive rate, FPR: false positive rate.


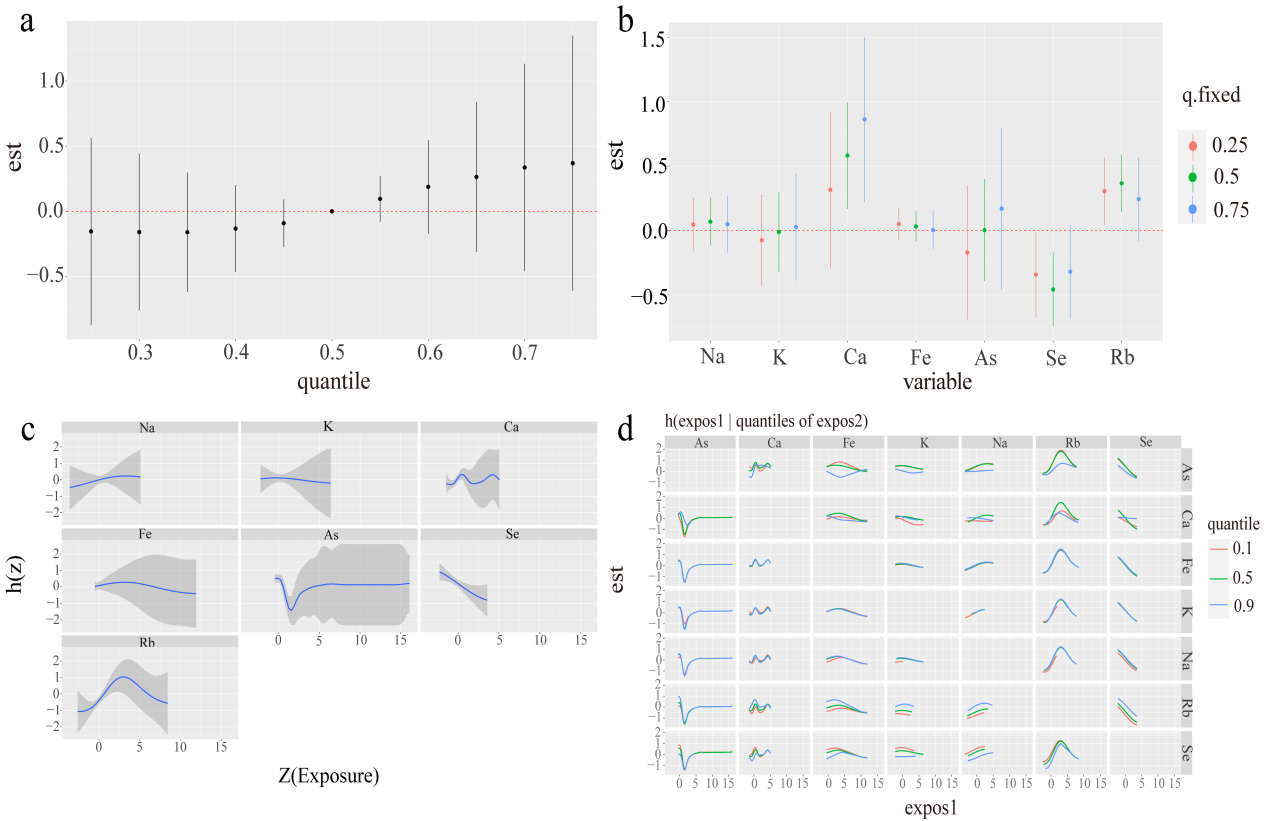
**FIGURE S7** Associations between blood metal mixtures and creatinine abnormalities among the study population by BKMR model. Model adjust for Sex and other laboratory indicators by using Spearman correlation. (a) The cumulative effect of the blood metal mixtures (estimates and 95% credible intervals). Metal mixtures are at a particular percentile (X-axis) compared to when exposures are all at 50th percentile. (b) The single-exposure effect (estimates and 95% credible intervals). (c) Univariate exposure-response functions and 95% confidence bands for each blood metal with the other mixtures fixed at the median. (d) Multiple exposure-response functions for: the other metal when one metal fixed at either the 25th, 50th, or 75th percentile and the test of metal mixtures is fixed at the median.


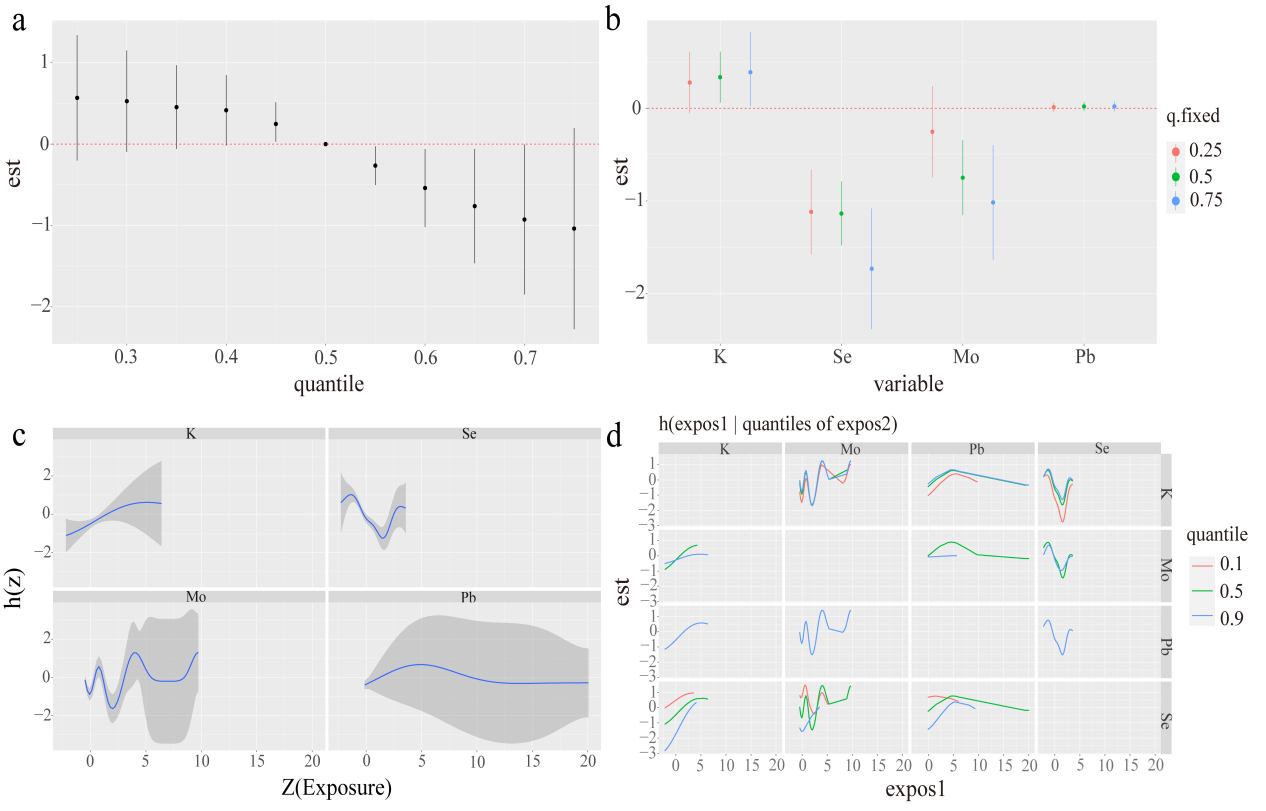
**FIGURE S8** Associations between blood metal mixtures and cystatin C abnormalities among the study population by BKMR model. Model adjust for Sex, Age and other laboratory indicators by using Spearman correlation. (a) The cumulative effect of the blood metal mixtures (estimates and 95% credible intervals). Metal mixtures are at a particular percentile (X-axis) compared to when exposures are all at 50th percentile. (b) The single-exposure effect (estimates and 95% credible intervals). (c) Univariate exposure-response functions and 95% confidence bands for each blood metal with the other mixtures fixed at the median. (d) Multiple exposure-response functions for: the other metal when one metal fixed at either the 25th, 50th, or 75th percentile and the test of metal mixtures is fixed at the median.

**TABLE S1** Description of CKD, creatinine and cystatin C abnormalities with general and clinical data.

| Variables | CKD | | | P(CKD) | Creatine | | P(Creatine) | CystatinC | | P(CystatinC) |
| --- | --- | --- | --- | --- | --- | --- | --- | --- | --- | --- |
|  | Case | | Control |  | Abnormal | Normal |  | Abnormal | Normal |  |
| Gender |  | |  | <0.001 |  |  | <0.001 |  |  | <0.001 |
| Female | 168 | | 68 |  | 106 | 128 |  | 126 | 108 |  |
| Male | | 282 | 27 |  | 259 | 50 |  | 248 | 61 |  |
| Age | 53(38,62) | | 37(32,49.5) | <0.001 | 52.5(38,62) | 43(34,57) | <0.001 | 54(41,63) | 39(31,50.5) | <0.001 |
| Estimated Glomerular Filtration Rate | 51.3(20.25,91.2) | | 111.4(103.2,119.1) | <0.001 | 39.55(15.48,70.45) | 110.53(99.33,119.93) | <0.001 | 40.7(15.6,73) | 111(102.8,120.1) | <0.001 |
| Potassium ion(mmol/L) | 4.2(3.89,4.61) | | 4.08(4,4.2) | 0.002 | 4.29(3.99,4.72) | 4.01(3.84,4.2) | <0.001 | 4.26(3.93,4.72) | 4.06(3.91,4.2) | <0.001 |
| Sodium ion(mmol/L) | 141.7(140.1,143.1) | | 144.1(142.4,144.1) | <0.001 | 141.7(140,143.4) | 142.6(140.68,144.1) | <0.001 | 141.6(140,143.1) | 143(140.9,144.1) | <0.001 |
| chloride ion(mmol/L) | 106.7(103.2,109.5) | | 107(105.9,107.7) | 0.201 | 106.6(102.78,109.5) | 107.1(105.9,108.2) | 0.085 | 106.7(102.7,109.8) | 107(105.9,107.7) | 0.254 |
| bicarbonate ion(mmol/L) | 24.5(22.3,26.6) | | 27.2(26.3,27.3) | <0.001 | 24.2(21.98,26.4) | 26.4(24.5,27.3) | <0.001 | 24.2(21.9,26.4) | 26.4(24.5,27.3) | <0.001 |
| Anion gap(mmol/L) | 14.51(12.49,17.48) | | 14.89(13.4,15.21) | 0.295 | 14.99(12.88,18.4) | 14.18(12.25,15.11) | <0.001 | 14.88(12.6,18.49) | 14.52(12.66,15.21) | <0.001 |
| calcium ion (mmol/L) | 2.2(2.03,2.31) | | 2.35(2.18,2.37) | <0.001 | 2.2(2.04,2.32) | 2.24(2.11,2.35) | 0.019 | 2.19(2.02,2.31) | 2.29(2.15,2.37) | <0.001 |
| Phosphorus ion(mmol/L) | 1.25(1.07,1.45) | | 1.2(0.99,1.3) | <0.001 | 1.28(1.08,1.51) | 1.18(1,1.3) | <0.001 | 1.28(1.09,1.5) | 1.18(1,1.3) | <0.001 |
| magnesium ion(mmol/L) | 0.91(0.84,1.01) | | 0.91(0.88,0.98) | 0.51 | 0.94(0.86,1.04) | 0.89(0.83,0.94) | <0.001 | 0.92(0.85,1.04) | 0.9(0.85,0.95) | <0.001 |
| Serum Albumin | 36.1(26.7,40.45) | | 43.8(42.4,45.4) | <0.001 | 36.6(29.18,41.2) | 40.8(33.43,43.85) | <0.001 | 36(26.6,40.2) | 42.4(38.5,45) | <0.001 |
| Leukocyte count | 6.87(5.56,8.32) | | 5.59(4.62,6.71) | <0.001 | 6.93(5.61,8.34) | 5.94(4.97,7.29) | <0.001 | 6.94(5.57,8.45) | 5.96(4.9,7.11) | <0.001 |
| Granulocyte Ratio | 65.2(57.4,71.8) | | 54.7(50,60.45) | <0.001 | 66.15(57.38,73.3) | 58.4(52.3,64.25) | <0.001 | 66.4(58.9,73.3) | 56.8(51.3,63) | <0.001 |
| Lymphocyte Ratio | 24.7(17.5,31.3) | | 35.3(32.3,41.4) | <0.001 | 24.01±10.07 | 31.89±9.19 | <0.001 | 23.66±9.86 | 33.09±8.7 | <0.001 |
| Hemoglobin Concentration | 122.620±25.5539 | | 136.215±15.9645 | <0.001 | 120(103.75,142.25) | 133(125,140) | <0.001 | 119(103,138) | 135(128,148.5) | <0.001 |
| Platelet Count | 225(182,278.5) | | 234(202.5,266) | 0.289 | 222.5(178.5,277.25) | 235.5(202,277.25) | 0.012 | 222(179,276) | 239(202.5,281.5) | 0.006 |

**TABLE S2** Differences in metallic elements within different stages of chronic kidney disease (CKD).

| Parameters (μg/L) | Stage 1  (GFR 90 mL/min ) | Stage 2  (GFR 89-60 mL/min) | Stage 3  (GFR 59-30 mL/min) | Stage 4  (GFR 29-15 mL/min) | Stage 5 (GFR 15- mL/min) | *P* |
| --- | --- | --- | --- | --- | --- | --- |
| Creatine | 65.61±13.55^ABC^ | 106.12±102.12^DE^ | 145.48±37.53^AFG^ | 271.3±83.31^BDFH^ | 826.84±361.14^CEGH^ | 0^**^ |
| Cystatin C | 0.95±0.22^ABCD^ | 1.3±0.35^AEFG^ | 1.99±0.57^BEHI^ | 3.11±0.72^CFHJ^ | 6.12±1.74^DGIJ^ | 0^**^ |
| Li | 36.56±48.64 | 103.45±594.79 | 106.24±700.02 | 39.26±69.44 | 35.96±52.65 | 0.585 |
| Be | 2.29±3.05^A^ | 1.62±2.27^B^ | 2.12±2.74^C^ | 0.85±1.4^ACD^ | 2.63±3.49^BD^ | 0.001^**^ |
| B | 67.68±70.03^ABC^ | 68.84±68.37^DEF^ | 109.42±100.12^ADGH^ | 161.6±82.68^BEG^ | 166.99±110.89^CFH^ | 0^**^ |
| Na | 6857775.13±1281076.92 | 6300360.16±1398660.45^a^ | 6719701.75±605062.51 | 6573285.78±580704.38 | 7036527.37±1692415.71^a^ | 0.001^**^ |
| Mg | 13283.75±6621.29^AB^ | 17247.56±23786.1^ACD^ | 13298.31±6183.6^CE^ | 14133.13±3557.57^F^ | 22227.83±8183.96^BDEF^ | 0^**^ |
| Al | 3903.71±3884.57 | 2872.74±3684.45 | 3900.38±3772.69 | 3540.58±3526.18 | 3643.97±4189.06 | 0.372 |
| K | 740085.53±167387.53^a^ | 662079.23±235027.31^b^ | 712406.82±221505.48^c^ | 684953.84±135094.51^d^ | 843327.7±225405.97^abcd^ | 0^**^ |
| Ca | 7263.4±5623.52^abcd^ | 13820.93±9169.17^ae^ | 13356.72±4919.55^bfg^ | 18859.13±6242.7^cefh^ | 10582.77±6549.99^dgh^ | 0^**^ |
| V | 0.33±0.72^a^ | 0.38±0.93^b^ | 0.28±0.8^c^ | 0.82±1.47^abc^ | 0.32±0.85 | 0.003^**^ |
| Cr | 18.53±49.78 | 15.36±41.85 | 14.31±23.04 | 25.54±19.37 | 25.67±61.94 | 0.255 |
| Mn | 21.7±109.62 | 23.19±67 | 13.77±45.11 | 20.38±25.01 | 29.07±140.83 | 0.847 |
| Fe | 1521.73±1898.06 | 1556.26±1777.84 | 1868.62±4540.73 | 1382.2±1786.23 | 1720.67±4676.31 | 0.888 |
| Co | 0.49±0.96^A^ | 8.29±36.78^ABCD^ | 0.53±1.01^B^ | 1.45±6.03^C^ | 0.61±0.97^D^ | 0.004^**^ |
| Ni | 8.57±38.67 | 25.13±104.53^ABC^ | 3.2±4.87^A^ | 3.72±17.79^B^ | 4.87±7.05^C^ | 0.022^*^ |
| Cu | 637.86±305.33^A^ | 587.71±290.48^B^ | 574.13±238.49^C^ | 666.35±140.82^D^ | 883.12±500.5^ABCD^ | 0^**^ |
| Zn | 2224.16±3033.36^AB^ | 481.23±546.77^ACDE^ | 2424.48±3709.42^CFG^ | 1514.18±1568.1^DFH^ | 3481.23±3359.75^BEGH^ | 0^**^ |
| Ga | 0.04±0.15 | 0.04±0.2 | 0.03±0.12 | 0.02±0.13 | 0.01±0.06 | 0.522 |
| As | 0.5±1.33^ABC^ | 1.13±1.52^DE^ | 2.16±3.2^A^ | 2.97±7.82^BD^ | 2.77±3.46^CE^ | 0^**^ |
| Se | 51.9±18.85^AB^ | 57.47±27.1^C^ | 51.6±17.29^DE^ | 41.3±15.57^ACDF^ | 59.12±21.64^BEF^ | 0^**^ |
| Rb | 159.78±52.46 | 165.04±75.12 | 176.45±82.16 | 166.78±43.95 | 153.49±65.74 | 0.172 |
| Sr | 28.29±39.44^abcd^ | 44.96±28.48^aefg^ | 66.34±42.48^be^ | 77.92±31.97^cf^ | 81.43±43.73^dg^ | 0^**^ |
| Mo | 16.31±30.87^AB^ | 3.38±4.33^ACD^ | 12.7±19.15^CE^ | 8.48±9.62^BF^ | 21.84±26.53^DEF^ | 0^**^ |
| Ag | 0.64±2.05 | 0.44±2.63 | 0.18±1.1 | 0.26±0.71 | 0.4±2.12 | 0.471 |
| Cd | 0.14±0.21 | 0.08±0.17^a^ | 0.09±0.16^b^ | 0.12±0.22 | 0.18±0.25^ab^ | 0.014^*^ |
| Sb | 18.65±20.84^A^ | 20.81±20.33^B^ | 21.53±21.28^C^ | 15.49±16.68 | 11.52±16.58^ABC^ | 0.004^**^ |
| Cs | 0.39±0.52^AB^ | 0.46±0.29^CD^ | 0.63±0.54^ACEF^ | 0.79±0.32^BDEG^ | 0.48±0.3^FG^ | 0^**^ |
| Ba | 30.39±34.24^A^ | 20.74±19.11^BC^ | 38.11±54.94^BD^ | 35.19±24.69^CE^ | 18.87±37.28^ADE^ | 0.001^**^ |
| Tl | 0.03±0.08 | 0.03±0.08 | 0.04±0.23 | 0.01±0.04 | 0±0.01 | 0.262 |
| Pb | 8.56±13.08 | 5.05±13.77 | 7.39±60.14 | 40.26±281.03 | 11.39±16.93 | 0.311 |
| U | 0.06±0.13^A^ | 0.07±0.09^B^ | 0.05±0.1^C^ | 0.12±0.11^ABCD^ | 0.04±0.05^D^ | 0^**^ |

**Correlation is significant at the 0.01 level (two tailed).

*Correlation is significant at the 0.05 level (two tailed).

Pairwise comparison between groups:

Different letters (a,b,c,d,e,f,g,h,i,j) indicate differences using LSD tests between groups(p<0.05), the same or no letters mean that there is no difference between groups(p>0.05) or can't be expressed in letters.

Different letters (A,B,C,D,E,F,G,H,I,J) indicate differences using Dunnett's tests between groups(p<0.05), the same or no letters mean that there is no difference between groups(p>0.05) or can't be expressed in letters.

**TABLE S3** Multivariate analysis of the association of creatinine abnormalities and concentration changes (95%CI) in blood metals.

| Stratification | Predictor Variables | Model 1 | | Model 2 | |
| --- | --- | --- | --- | --- | --- |
|  |  | Exp(β)(95%CI) | P(Creatine) | Exp(β)(95%CI) | P(Creatine) |
| Total | B | 1.006(1.003,1.01) | 0 | - | 0.52 |
|  | Mg | 1.0016(1.0002,1.003) | 0.022 | - | 0.165 |
|  | K | 1.002(1.001,1.003) | 0 | - | 0.918 |
|  | Cu | 0.998(0.997,0.999) | 0 | - | 0.213 |
|  | Se | 0.98(0.97,0.99) | 0.002 | - | 0.794 |
|  | Sr | 1.02(1.01,1.02) | 0 | - | 0.855 |
|  | Cs | 2.55(1.31,4.95) | 0.006 | - | 0.634 |
|  | U | 0.02(0,0.2) | 0.001 | - | 0.718 |
| Sex |  |  |  |  |  |
| Male | B | 1.01(1.01,1.02) | 0 | - | 0.87 |
|  | Sr | 1.02(1.01,1.03) | 0.002 | - | 0.976 |
|  | Mo | 0.97(0.95,0.99) | 0 | - | 0.943 |
|  | Na | - | 0.637 | 1(1,1) | 0.774 |
|  | K | - | 0.849 | 1(1,1) | 0.87 |
|  | Ca | - | 0.852 | 1(0.98,1.02) | 0.929 |
|  | Fe | - | 0.214 | 1.01(0.9,1.14) | 0.815 |
| Female | B | 1.01(1,1.01) | 0.002 | - | 0.278 |
|  | Mg | 1(1,1) | 0.001 | - | 0.221 |
|  | K | 1(1,1) | 0.019 | - | 0.629 |
|  | Se | 0.98(0.96,1) | 0.031 | 0.96(0.93,0.99) | 0.013 |
|  | Sr | 1.03(1.02,1.04) | 0 | - | 0.485 |
|  | Mo | 1.02(1,1.04) | 0.021 | - | 0.101 |
|  | Pb | 1(1,1.01) | 0.13 | - | 0.768 |
|  | As | - | 0.243 | 0.59(0.41,0.84) | 0.003 |
|  | Rb | - | 0.902 | 1.028(1.004,1.052) | 0.021 |

Model 1: adjusted for metals for univariate analyzes.

Model 2: model 1 plus sociodemographic and general blood indicators for univariate analyzes.

**TABLE S4** Multivariate analysis of the association of cystatin C abnormalities and concentration changes (95%CI) in blood metals.

| Stratification | Predictor Variables | Model 1 | | Model 2 | |
| --- | --- | --- | --- | --- | --- |
|  |  | Exp(β)(95%CI) | P(Cystatin C) | Exp(β)(95%CI) | P(Cystatin C) |
| Total | B | 1.004(1.001,1.007) | 0.024 | - | 0.174 |
|  | K | 1.0004(1.0003,1.0005) | 0 | 1.004(1.002,1.005) | 0 |
|  | Se | 0.97(0.96,0.98) | 0 | 0.98(0.96,0.99) | 0.002 |
|  | Sr | 1.01(1.01,1.02) | 0 | - | 0.459 |
|  | Mo | - |  | 1.015(1.003,1.027) | 0.016 |
|  | Pb | - |  | 1.004(1.001,1.008) | 0.022 |
| Sex |  |  |  |  |  |
| Male | K | 1.004(1.003,1.006) | 0 | 1.005(1.002,1.007) | 0 |
|  | Se | 0.97(0.96,0.99) | 0 | 0.97(0.95,0.99) | 0.007 |
|  | Sr | 1.02(1.01,1.03) | 0 | - | 0.767 |
|  | Mo | - | 0.541 | 1.019(0.998,1.04) | 0.075 |
| Female | B | 1.007(1.002,1.012) | 0.009 | - | 0.501 |
|  | K | 1.004(1.002,1.006) | 0 | - | 0.225 |
|  | Se | 0.98(0.96,0.99) | 0.004 | 0.97(0.95,0.99) | 0.007 |
|  | Rb | 0.984(0.973,0.995) | 0.004 | - | 0.731 |
|  | Sr | 1.02(1.01,1.03) | 0.001 | - | 0.362 |
|  | Pb | 1.002(0.999,1.006) | 0.153 | 1.004(1,1.007) | 0.058 |

Model 1: adjusted for metals for univariate analyzes.

Model 2: model 1 plus sociodemographic and general blood indicators for univariate analyzes.

**TABLE S5** Summary of the prediction results with different models including CKD disease, creatinine and cystatin C abnormalities.

| Model | Series | Parameters | cv.fold | Training set | | |  | Test set | | |
| --- | --- | --- | --- | --- | --- | --- | --- | --- | --- | --- |
|  |  |  |  | RMSE | R^2^ | MAE |  | RMSE | R^2^ | MAE |
| SVM (Radial) | CKD | cost=1,gamma=0.1 | 10 | 0.085 | 0.947 | 0.036 |  | 0.097 | 0.942 | 0.041 |
| SVM (Radial) | Creatine | cost=1,gamma=0.1 | 10 | 0.226 | 0.773 | 0.142 |  | 0.257 | 0.693 | 0.183 |
| GPR (Laplacedot) | Cystatin C | sigma = 0.4909 | 10 | 0.198 | 0.825 | 0.114 |  | 0.253 | 0.704 | 0.137 |

**TABLE S6** Posterior inclusion probabilities (PIPs) for metal mixtures into different groups models, using the Bayesian kernel machine regression (BKMR) model.

| Series | Parameters | Metals | PIPs |
| --- | --- | --- | --- |
| CKD | r_1_=0.044, β_1_=2.7, sigsq.eps=1 | Na | 1 |
|  |  | Mg | 1 |
|  |  | K | 1 |
|  |  | Ca | 1 |
|  |  | Cu | 1 |
|  |  | Se | 1 |
|  |  | Ag | 1 |
| Creatine | r_1_=0.026, β_1_=2, sigsq.eps=1 | Ca | 1 |
|  |  | As | 1 |
|  |  | Rb | 1 |
|  |  | Fe | 0.968 |
|  |  | Se | 0.876 |
|  |  | Na | 0.848 |
|  |  | K | 0.796 |
| Cystatin C | r_1_=0.025, β_1_=1.2, sigsq.eps=1 | Se | 1 |
|  |  | Pb | 1 |
|  |  | K | 0.99 |
|  |  | Mo | 0.964 |
